# A survey of crystallographic quality metrics from CIFs in the Cambridge Structural Database

**DOI:** 10.1107/S2052252525007134

**Published:** 2025-09-22

**Authors:** Clare A. Tovee, Seth B. Wiggin, Natalie T. Johnson, Philip I. Andrews, Matthew P. Lightfoot

**Affiliations:** a Cambridge Crystallographic Data Centre 12 Union Road CambridgeCB2 1EZ United Kingdom; Sun Yat-Sen University, China

**Keywords:** Cambridge Structural Database, CSD, crystal structures, data quality, *checkCIF*, *R* factors, residual electron densities, goodness of fit, data resolution, parameter shifts

## Abstract

The Cambridge Structural Database (CSD) is a comprehensive repository of over 1.3 million unique crystallographic datasets, compiled from data deposited at the Cambridge Crystallographic Data Centre (CCDC). Given the vast size of this collection, identifying datasets suitable for reuse in a particular area of study is critical. Traditionally, the *R* factor has been used as an indicator of structure refinement quality; however, numerous additional metrics can provide valuable insights into the refinement and structural model. Some of these metrics have been extracted from data in the deposited Crystallographic Information Files (CIFs) and added to the CSD. This study surveys these selected crystallographic data items and explores the variability in them between different types of structures. Through a statistical analysis of their values, this work aims to assist with the selection of relevant structures for future reuse.

## Abstract 2

This study examines data extracted from Crystallographic Information Files (CIFs) deposited at the Cambridge Crystallographic Data Centre (CCDC) to gain insight into patterns and trends seen in the quality of structural models obtained from structure refinements. From the results of over 1 300 000 individual datasets, this work aims to show what can be considered as typical values over a variety of experimental conditions.

## 1. Introduction

Whilst crystallographic structure solution is a technique with many facets and nuances, the fundamental process remains the same. Diffraction data are collected, and the crystallographer then derives a model for the structure that best fits the measured data. The most widely used measure to describe the fit of the model to the data is the *R* factor, which refers to the difference in the structure-factor amplitudes between the observed and calculated values for the refinement; however, there are many other metrics that can be used to give an insight into the validity of the structural model proposed, which in this paper we will refer to collectively as ‘quality metrics’.

The use of quality metrics to assess the crystallographic model proposed by the crystallographer has always been an important factor for evaluating the potential uses of the data and the conclusions that might be drawn from them. This evaluation of data quality is particularly important for the use of large collections of data such as the Cambridge Structural Database (CSD). A common use of such databases is ‘data mining’ – where insights or evidence might be obtained by using many data sources. Indeed, this was one of the motivations for the establishment of the CSD in 1965; the founder of the CSD, Dr Olga Kennard, when recounting a discussion with J. D. Bernal, stated ‘We had a passionate belief that the collective use of data will lead to the discovery of new knowledge, which transcends the results of individual experiments’ (Kennard, 1997 ▸). Over the course of the CSD’s 60-year history, numerous discoveries have been made through the mining of the data, including proof of hydrogen bonds (Taylor & Kennard, 1982 ▸), insights into ring geometry (Allen, 1982 ▸) and the Bürgi–Dunitz angle (Rodríguez *et al.*, 2023 ▸).

For crystallographic analysis, data evaluation occurs throughout the refinement process. Refinement software gives indications of the standard of the refinement, such as the colour-coded GUI of *Olex2* (https://www.olexsys.org/olex2/docs/getting-started/getting-around-olex2/introduction/), whilst publication of crystallographic data often requires the author to use the IUCr’s *checkCIF* service (Spek, 2009 ▸; Spek, 2020 ▸) prior to submission to ensure that the data are of the required standard. This is not to suggest that structure validation is a solved problem: the applicability and weaknesses of some metrics remain topics of discussion (Henn, 2019 ▸), as does the reliability of published data (Raymond & Girolami, 2023 ▸). Structure validation is discussed in detail, with particular reference to powder diffraction, in Volume H of *International Tables for Crystallography* (Kaduk, 2019 ▸). The *checkCIF* service is also expanding to include more tailored checks for different data items to reflect the ever-changing range of tools and techniques available (McMahon, 2021 ▸; Aragon *et al.*, 2024 ▸).

The Cambridge Crystallographic Data Centre (CCDC) currently offers a variety of tools to help the user to prepare a suitable dataset for analysis. These range from the integration of the IUCr’s *checkCIF* service during the CCDC and FIZ Karslruhe joint deposition service, the *Mogul* software to help assess the molecular geometry of a structure, through to services for publishers and referees during the peer-review process. The CCDC software portfolio contains a number of data subsets (van de Streek, 2006 ▸; Moghadam *et al.*, 2017 ▸; Moreno, 2021 ▸) with the aim of providing the user with a smaller selection of structural data for particular use cases. This can be beneficial, as the CSD aims for as complete as possible coverage of the literature and attempts to report objectively what is presented, rather than imposing overly strict corrections and standardization. There are also a variety of filtering options to allow the user to select their own bespoke subset of data. The current filtering options include those based on chemistry (*e.g.* searching by substructure), refinement (*e.g.R* factor, disorder), type of study (X-ray, electron or neutron diffraction) and on chemical properties (*e.g.* bioactivity).

The range of fields from which the user can assess data has been enhanced in the CCDC’s 2025.1 software release. The enhancements include publication titles, new calculated data related to porous materials and additional quality metrics taken from the CIFs deposited at the CCDC. In this article we aim to explore the original deposited CIFs from which the new quality metrics in the CSD have been made available, to give an insight into how these fields might be useful to researchers.

## 2. Methodology

The data for this survey were extracted from the CCDC’s CIF archive, analogous to structures in the CSD at the time of the 2024.3 release, with a total of 1 086 480 files analysed. This corresponded to approximately 80% of the total size of the CSD and included almost all entries from the time when CIFs became routinely deposited. The types of structures analysed were determined using the CSD Python API and the statistical analysis was carried out with Python.

This study focused on single-crystal data items from the Core CIF dictionary (https://www.iucr.org/resources/cif/dictionaries/cif_core). The CSD also contains structure solutions from powder X-ray diffraction (PXRD). This category of structures, however, makes up a very small proportion of the whole CSD (0.7%) and as PXRD uses additional, different CIF items and descriptors (see the powder CIF dictionary at https://www.iucr.org/__data/iucr/cifdic_html/1/cif_pd.dic/index.html) to assess the quality of the refinement, this might mean that the values are not directly comparable to those from single-crystal studies. It was therefore decided that structures derived from PXRD were out of the scope of this investigation.

### 2.1. Selection of CIF items for analysis

The aim of this study was to gain insight into what values could be considered as ‘normal’ in terms of crystallographic data quality within the CSD, which could then help users to judge the reliability individual structures. We hoped to achieve this by investigation of the seven CIF data items listed below, plus data resolution, as although this value is very rarely reported in a CIF it has been calculated separately from the data available.

_refine_ls_R_factor_gt

_refine_ls_wR_factor_ref

_refine_ls_shift/su_max

_refine_diff_density_max

_refine_diff_density_min

_refine_ls_goodness_of_fit_ref

_diffrn_reflns_theta_max

These CIF data items were chosen for several reasons. Analysis of the CIFs deposited at the CCDC showed that the items were commonly included, and the values given were easily interpretable with minimal additional curation needed. Each item gives an insight into either the data collection or refinement process. A further consideration is that each of these data items can be used to make comparisons between different data collections. It is important to note that these data items focus on quality metrics from the perspective of the refinement as opposed to the ‘chemical suitability’ of the resulting structural model. Other spectroscopic techniques and the CCDC’s *Mogul* (Bruno *et al.*, 2004 ▸) and *Isostar* (Bruno *et al.*, 1997 ▸) software can be used to aid an assessment of the ‘chemical correctness’ of the structure. It can also be assumed that the peer-review process will ensure that the proposed structural model is reasonable, but in theory a refinement with good-quality metrics could be produced by creating a ‘chemically impossible’ structure.

Values for three additional CIF items were also collected: the numbers of parameters, restraints and constraints used in a refinement, as reported in _refine_ls_number_parameters, _refine_ls_number_restraintsand _refine_ls_number_constraints, respectively. These values are of benefit when assessing the quality of an individual structure, or potentially when comparing one structure to another; however, a statistical analysis of the collected data is unlikely to give any meaningful result. As such, these attributes are not discussed further in this article, but are reported in the supporting information.

Additional CIF items that might help with the assessment of structure quality, such as _refine_special_details, were not selected. Although these might contain valuable information from the crystallographer, they can contain data as free text, which precludes a statistical analysis. There are several other attributes of the refinement that were judged to be beyond the scope of this study but might provide further interesting insights. These include data completeness, which would be of particular interest for data collections at non-ambient pressure, and details of the weighting scheme used in the refinement (Henn, 2025 ▸).

### 2.2. Selection of categories

Since its inception in 1965, the CSD has contained a diverse range of structures, broadly covering small-molecule organic and metal–organic structures, with defined boundaries for inorganic data with the ICSD (Zagorac *et al.*, 2019 ▸) and macromolecular data held by the Protein Data Bank (PDB) (Berman *et al.*, 2000 ▸). This presents a challenge when considering a ‘typical’ CSD structure; the structure could range from a small organic molecule to a huge metal–organic complex. The data collection and refinement techniques also have a wide variety of possibilities. In this survey of quality metrics, we have chosen a series of categories with which to group structures. The selection is more illustrative than definitive; the primary considerations being that they should be easy to define, so that all structures can be unambiguously allocated to a category, and that the categories are sufficiently different so that we might reasonably expect an impact on the quality metrics observed.

The categories we selected were:

(1) Refinement type – independent atom model (IAM) or aspherical atom refinement.

(2) Disordered structures (using the CSD definition of having multi-site disorder of non-hydrogen atoms with partial occupancy atoms, symmetry disorder or unmodelled atoms, *e.g.* using *SQUEEZE* or *MASK*) (Spek, 2015 ▸).

(3) Organic or metal–organic structures (using the CSD definition of ‘organic’ as any structure that does not contain a transition metal, lanthanide, actinide or any of Al, Ga, In, Tl, Ge, Sn, Pb, Sb, Bi, Po).

(4) Polymeric or discrete structures.

(5) Ambient or non-ambient pressure.

(6) Radiation source (either X-ray, neutron or electron).

### 2.3. Comparison with *checkCIF* values

The IUCr’s *checkCIF* tool is widely used to ensure the integrity, consistency and data completeness of a CIF prior to publication. For many journals, submission of a *checkCIF* report is a mandatory part of the submission process. Deposition of crystal structure data to the CCDC via the online CIF deposition and validation service optionally integrates the *checkCIF* service and encourages users to address any serious issues that are revealed. *checkCIF* currently contains a total of 572 data validation tests in its *checkCIF*/*PLATON* service.

*checkCIF* produces four levels of alert. Alert levels A to C highlight potential problems of decreasing severity: level A alerts indicate potentially serious problems, level B alerts suggest less serious potential problems, and level C alerts highlight deviations from typical values. Note that the alerts might correlate with each other, so whilst for example a single level C alert might not be significant, a series of such alerts might suggest a wider issue. *checkCIF* also produces level G alerts, and although these are more often informational in nature these might be important to ensure required metadata are present and to check that common default values have not been included erroneously.

This analysis of data deposited in the CCDC’s archive presents an opportunity to compare the values used by *checkCIF* to assign the level of an alert against published experimental data. However, as stated previously, care should be taken in this comparison because a refinement having typical values does not necessarily mean it is correct and vice versa.

### 2.4. Challenges

The statistical analysis also presents several challenges. Despite each category being large enough to minimize the impact of an individual data set, the categories have significantly different sizes. For example, for comparisons of pressure or radiation, the sample sizes differ by several orders of magnitude, therefore a direct comparison between them has limited validity. Structures might also belong to multiple categories, *e.g.* a disordered organic structure.

In addition, many statistical analysis methodologies assume that the data at least approximate a normal distribution. This assumption cannot be made for many of the data items being investigated, where skewed or multinomial distributions are more suitable descriptions. We can make preliminary comments, but further insights are out of the scope of this publication and a future direction for research in this area.

## 3. Results and discussion

### 3.1. Data completeness and outliers

The original publication describing the CIF states that ‘The Crystallographic Information File is a general, flexible and easily extensible free-format archive file; it is human and machine readable and can be edited by a simple text editor’ (Hall *et al.*, 1991 ▸). While this flexibility is advantageous, enabling widespread adoption and supporting a diverse range of applications, it also introduces challenges. These challenges include the potential for missing data and incorrect syntax, which complicate large-scale data analysis. Additionally, the use of free-text fields could also be problematic in this context.

**Figure 1:**
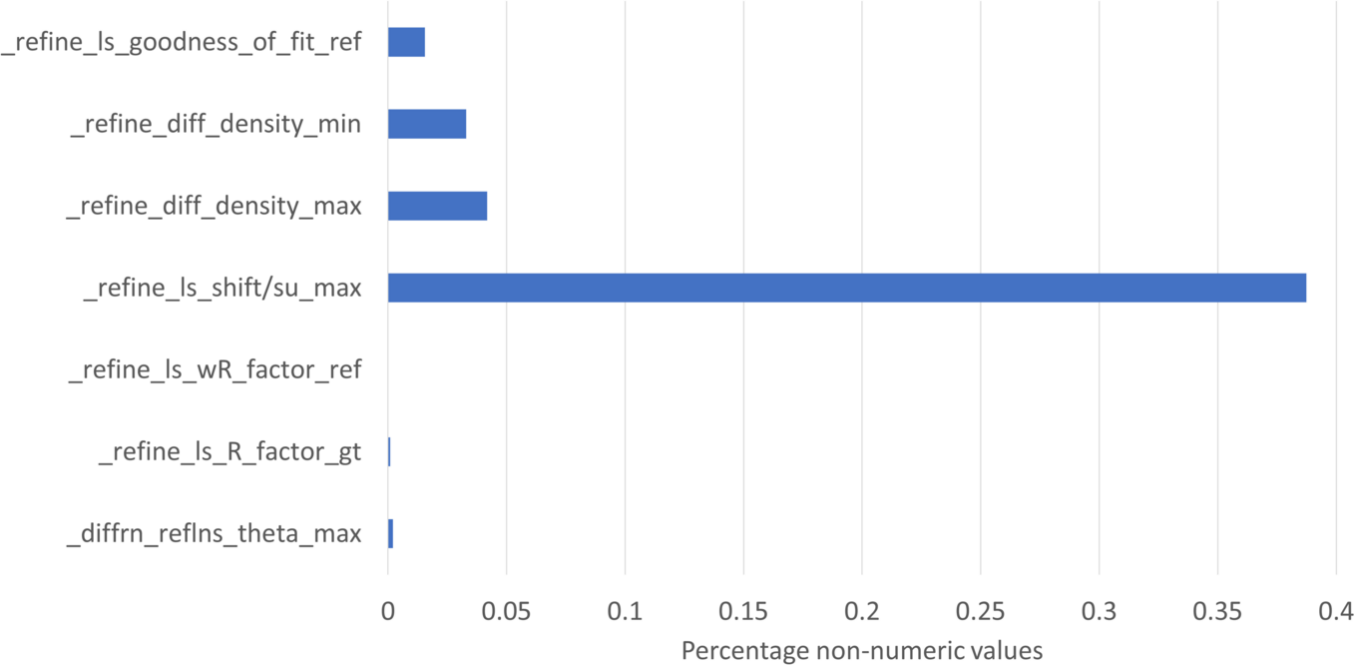
Chart of percentages of values that are non-numeric for the selected items.

Within the extensive dataset of the CSD there are inherent outliers, as expected in any data distribution. The two main challenges in processing the data were missing data and non-numeric data. The data items used in this study were found to be around 96–98% complete (see the supporting information). Instances of non-readable data were minimal, as indicated by the small percentage values shown in Fig. 1 ▸. Notably, the slightly higher percentage for _refine_ls_shift/su_max was due to entries reported as ‘less than’ some value (*e.g.* <0.01 or <0.001). These values were classified as ‘unreadable’ and were excluded from the analysis.

Further exclusions were made to prevent anomalous data from skewing the results. These included negative values (or positive values for _refine_diff_density_min) as well as values that were orders of magnitude larger than expected and were deemed to be invalid. Whether these discrepancies were due to issues with the refinement, syntax error or the use of units inconsistent with the CIF dictionary was not investigated further in this study. The ranges for these items are provided in Table 1 ▸; the ranges used have been deliberately chosen to exclude a very small number of examples where the value given is ‘impossible’. For example, an *R* factor above 100% is within the permitted range according to the CIF dictionary but does not correspond to a meaningful value. We have tried to avoid making stricter judgements on the range of values that could be considered ‘reasonable’ in order to provide the most faithful representation of the source data.

### 3.2. Analysis of CIF items

#### 3.2.1. *R* factors

Fundamentally, the process of a crystal-structure determination from diffraction data involves proposing a structural model that best fits the electron density derived from the diffraction data (Spek, 2009 ▸). Therefore, differences between the model and data can be used to measure the quality of a refinement.

**Figure 2:**
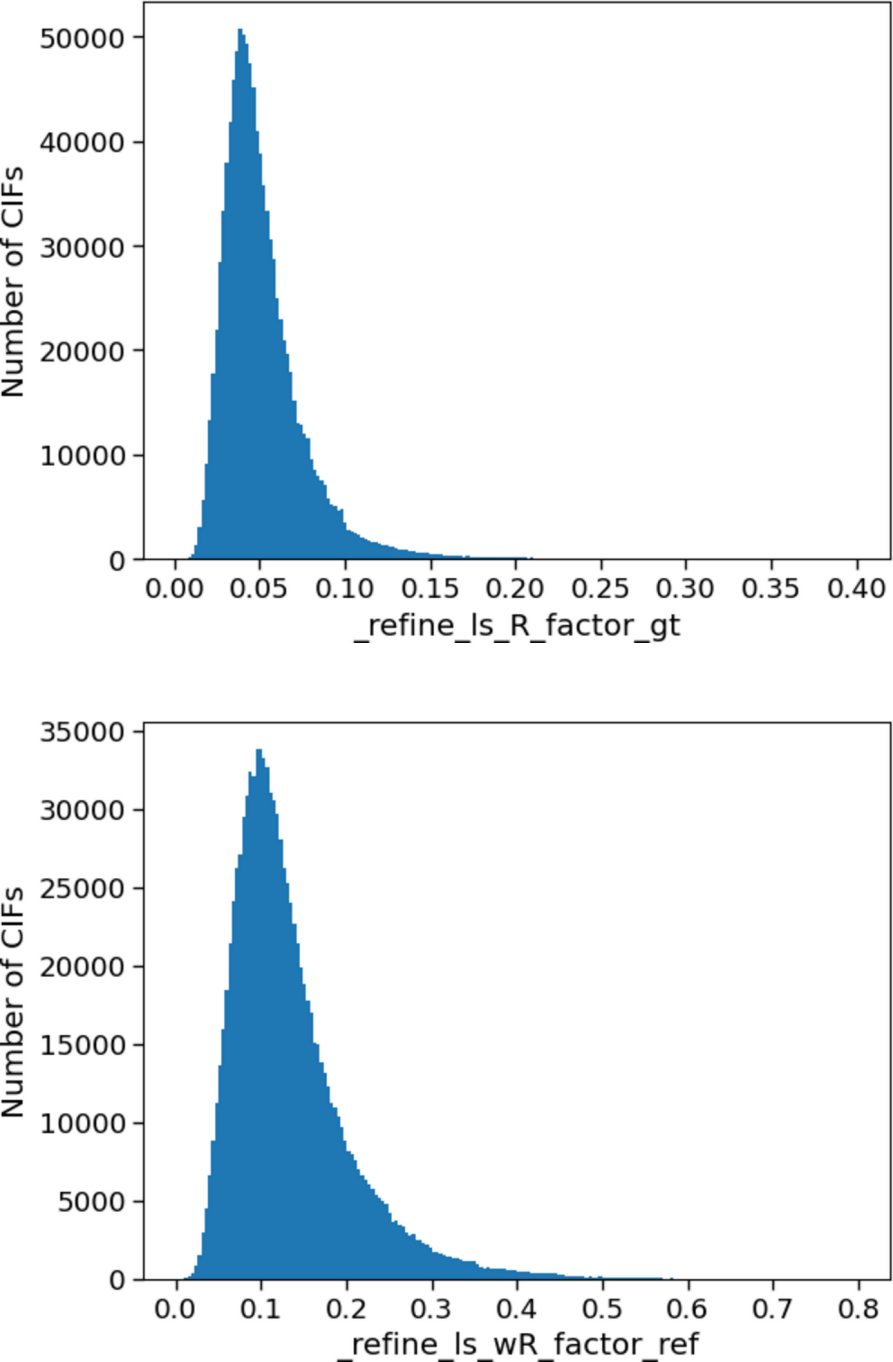
Histograms of _refine_ls_R_factor_gt and _refine_ls_wR_factor_ref for structures in the CSD.

One of the most commonly reported metrics for assessing refinement quality is the *R* factor, which represents how well the structural model in the CIF describes the measured reflection data. This value might be reported for all the measured reflection data, or just for those reflections with an intensity above a defined threshold. A related measure, the weighted residual factor (*wR* factor), is similar to the *R* factor but also considers the weighting scheme applied to the refinement. Fig. 2 ▸ shows the distribution of values for both items. In both cases, a low *R* factor is desired, as this would indicate that minimal residual density remains unaccounted for in the model. The *wR* factor typically has a higher value than the *R* factor, a trend reflected in the mean values reported in the CSD. Specifically, the reported mean values (and standard deviations) are 0.054 (0.025) for _refine_ls_R_factor_gt and 0.135 (0.072) for _refine_ls_wR_factor_ref. The median values for these items are slightly lower than the mean at 0.046 and 0.118 for _refine_ls_R_factor_gt and _refine_ls_wR_factor_ref, respectively. The completeness of the data for these items is 95.7% and 94.4% for _refine_ls_R_factor_gt and _refine_ls_wR_factor_ref, respectively, indicating that these items are well represented and that the dataset contains a large number of structures, which encompass a wide variety of chemistry in the CSD.

**Figure 3:**
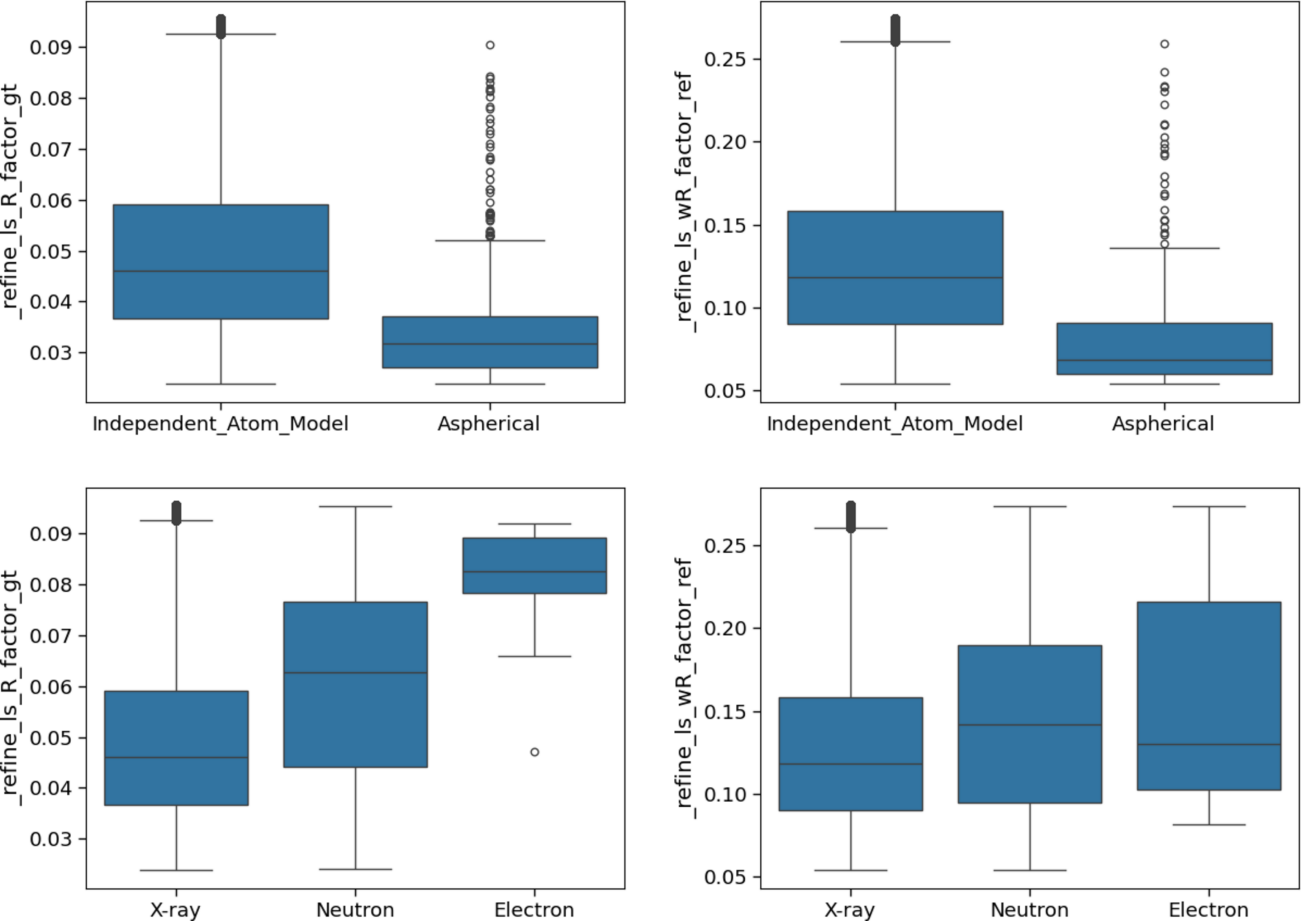
Box plots for *R* factor (left) and *wR* factor (right) by type of atom modelling (top) and radiation source (bottom).

In examining various categories of structures (Fig. 3 ▸), distinct trends emerged in the data. Some of these trends align with expectations; for example, aspherical atom refinement methods tend to yield lower *R* factors, as these methods provide a more accurate description of the electron density around atoms. Additionally, when considering the radiation source, *R* factors for electron diffraction are generally higher than those for X-ray and neutron diffraction due to the stronger interaction of electrons with the sample, which often results in multiple scattering of the electron beam by the sample. This effect can be mitigated by employing a dynamical scattering model in structure refinement, but this is not applied in every electron structure refinement (Palatinus *et al.*, 2015 ▸).

**Figure 4:**
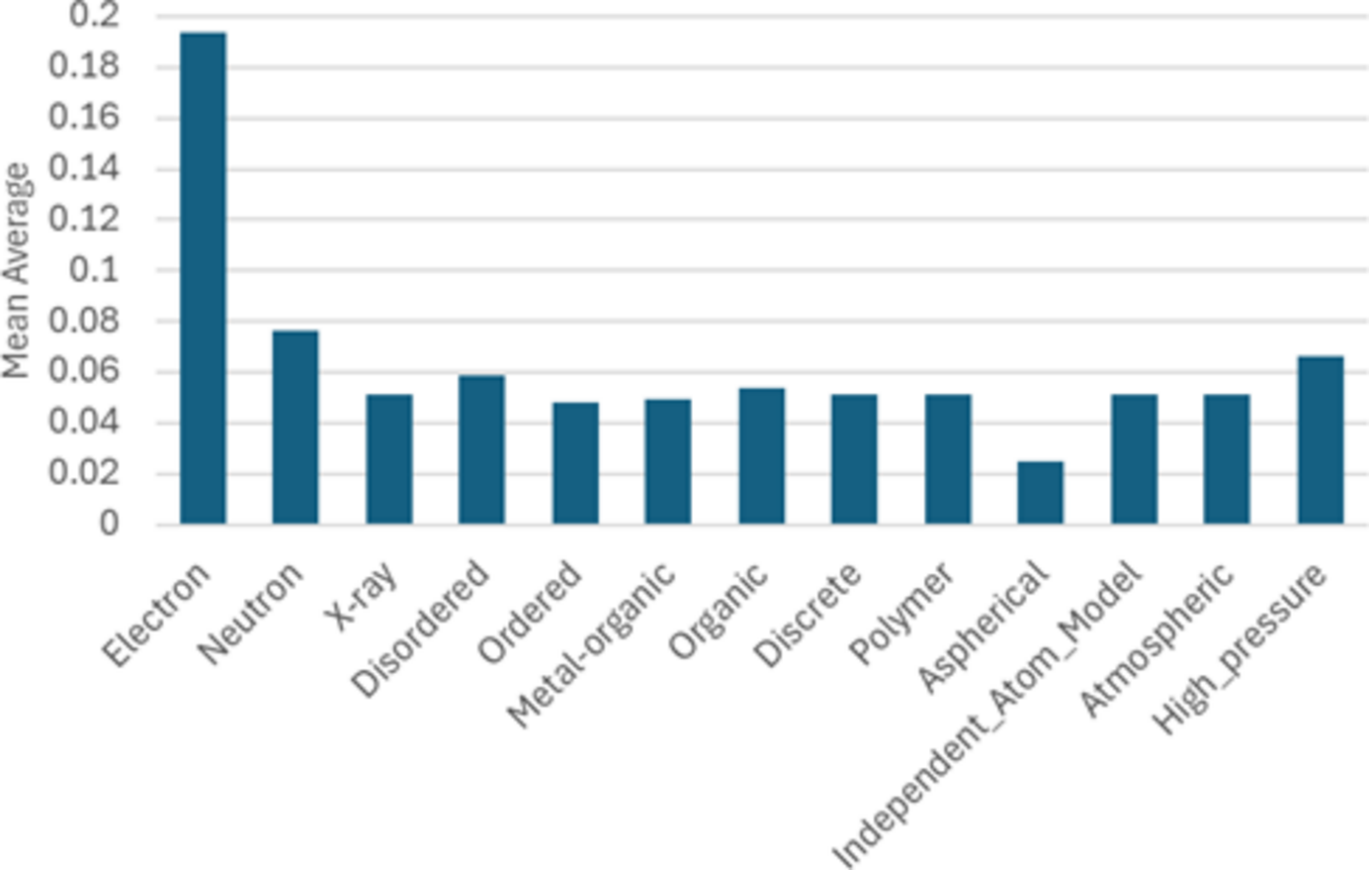
Bar chart of mean average of the *R* factor for each category.

While some factors, such as disorder or metal content, might intuitively influence the *R*-factor values (*e.g.* disorder potentially increasing the *R* factor if not all disorder is modelled, or metals contributing higher electron density), the observed differences remain relatively small. Specifically, comparing *R* factors across different data subsets – whether by mean, median or selected quantiles – reveals no significant differences in the trends (Fig. 4 ▸).

The lowest-level *checkCIF* C alerts for the *R* factor and *wR* factor are given for values above 0.1 and 0.25, respectively. Equivalent 90th percentile values for the CSD as a whole are 0.0804 and 0.227, suggesting that *checkCIF* alerts are well placed to highlight unusual values.

#### 3.2.2. Residual electron density

The difference or residual electron density is the density unmodelled by the structure solution (when subtracting the electron density calculated from the model from the measured electron density). The items _refine_diff_density_max and _refine_diff_density_min record, as given in the IUCr CIF dictionary (https://www.iucr.org/__data/iucr/cifdic_html/1/cif_core.dic/Irefine_diff_density_.html), ‘the largest and smallest values in electrons per ångström cubed, of the final difference electron density’.

For a theoretically perfect refinement, the value of _refine_diff_density_max would be zero – indicating that the proposed model completely accounts for all the measured electron density. In reality, all refinements will be left with residual noise in the electron density. Significant unaccounted-for electron density, however, is indicative of potential issues with the refinement (Spek, 2020 ▸).

**Figure 5:**
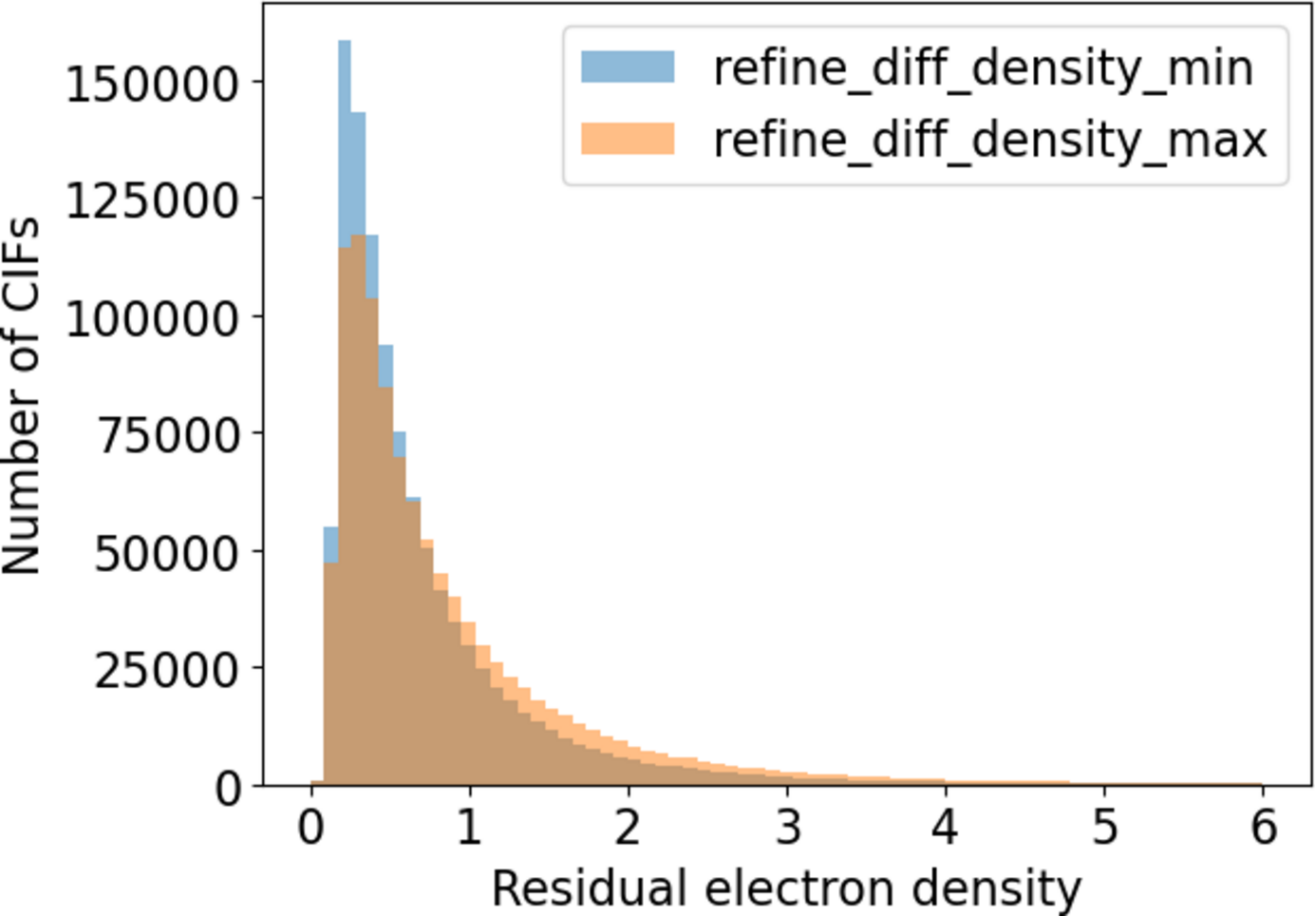
Distributions of _refine_diff_density_max and |_refine_diff_density_min| for crystal structures in the CSD.

Analysis of the structures deposited at the CCDC shows that the maximum and minimum electron density are well reported, with over 97% of CIFs investigated containing usable data; almost all the remaining CIFs did not contain the items, with a tiny proportion (around 0.04%) containing non-numeric data. The magnitudes of the values of the minimum electron density, *i.e.* |_refine_diff_density_min|, are shown in Table 2 ▸ and Fig. 5 ▸ in order to facilitate comparison with the maximum values. The overall mean values for _refine_diff_density_max and _refine_diff_density_min from the whole CSD were 0.904 and −0.714, with median values of 0.596 and −0.481 (Table 2 ▸). These values broadly agree with the received wisdom that the values are similar, as shown in Fig. 5 ▸. Although the minimum electron density has more values between zero and one, the maximum has slightly more larger values, which reflects the larger average value for _refine_diff_density_max.

The IUCr’s *checkCIF* service performs a series of checks of _refine_diff_density values. When assessing a suitable level of residual density, the test value it uses is defined as 10% of the largest atomic number (*Z*_max_) of an element in the structure (DTEST = 0.1 * ZMAX, see https://journals.iucr.org/services/cif/checking/DIFMN_02.html). For a maximum residual density of twice this value, *checkCIF* assigns an alert level A, indicating a potentially serious problem with the data and/or model. Level B and C alerts are given for residual density values greater than 10% and 7.5% of *Z*_max_, respectively. The 10% value appears to be an arbitrary, ‘common-sense’ choice; the authors are unaware of any previous systematic attempts to validate this against small-molecule experimental data. When looking at high-resolution data, Fukin *et al.* (2022 ▸) note ‘the residual ED [electron density] on heavy metal atoms (Sb, Pb, Ln) can reach up to 10 e Å^–3^’ – a value which considerably exceeds the 10% value used in the test.

**Figure 6:**
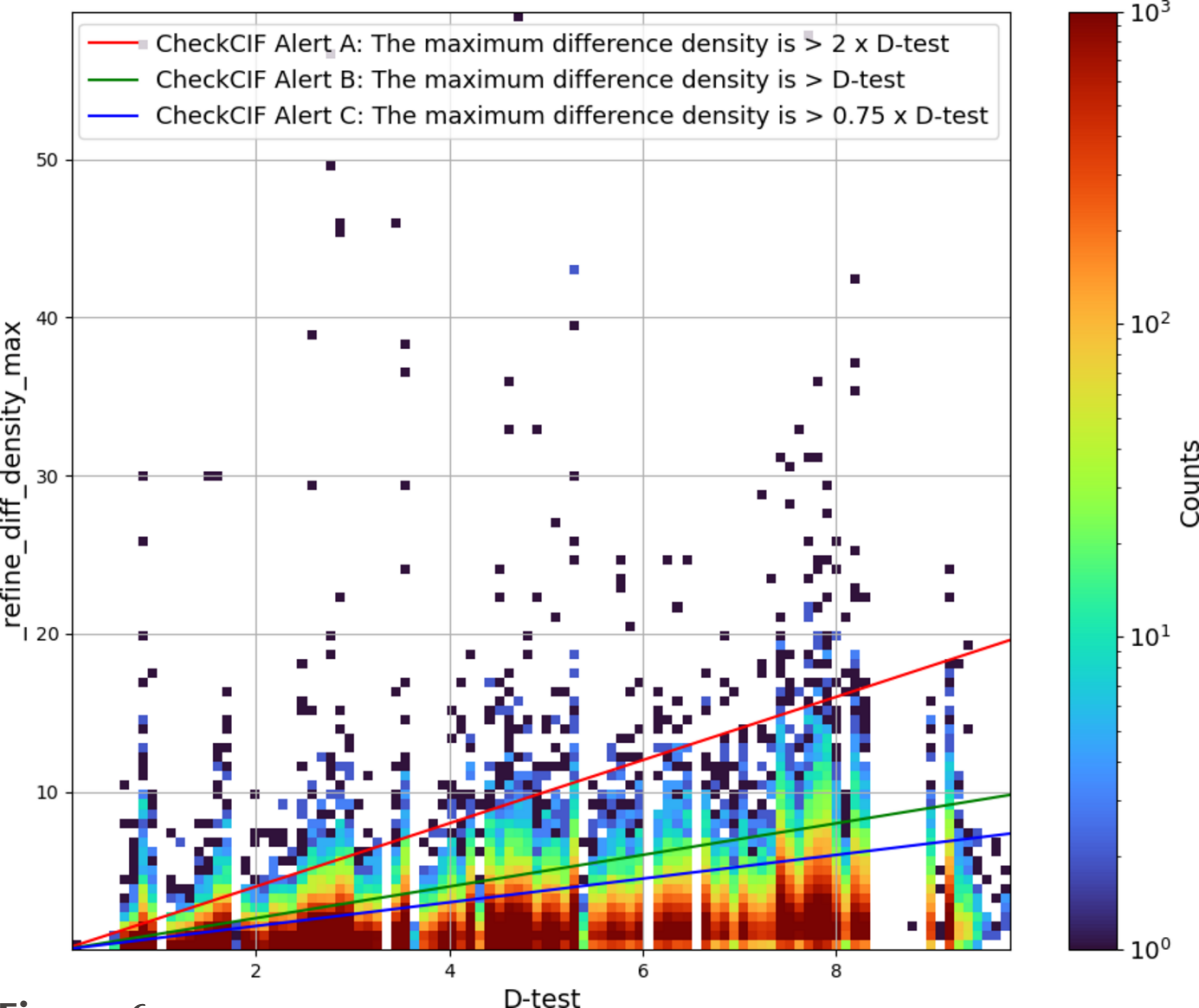
Scatter plot of the *checkCIF* DTEST value against _refine_diff_density_max with a log scale for the colour indicating the number of structures within each binned grid point.

To examine how well the ‘expected limits’ for residual density defined by *checkCIF* alerts apply to CSD data, the expected level of residual density according to the *checkCIF* DTEST value has been plotted against the experimentally measured values (Fig. 6 ▸). The plot includes lines to show the boundaries between *checkCIF*’s A, B and C alerts, based on the criteria mentioned above. It can be seen that when considering the CSD, which is a database of predominantly small-molecule organic compounds, for the majority of structures the 10% value appears to be a useful measure of quality. While Fig. 6 ▸ shows several data points well above the A alert level value, it should be noted that given the logarithmic scale used in the figure, these correspond to a very small number of structures from a total dataset of over a million values. The plot for the minimum residual density, which is included in the supporting information as Fig. S2, shows the same features.

**Figure 7:**
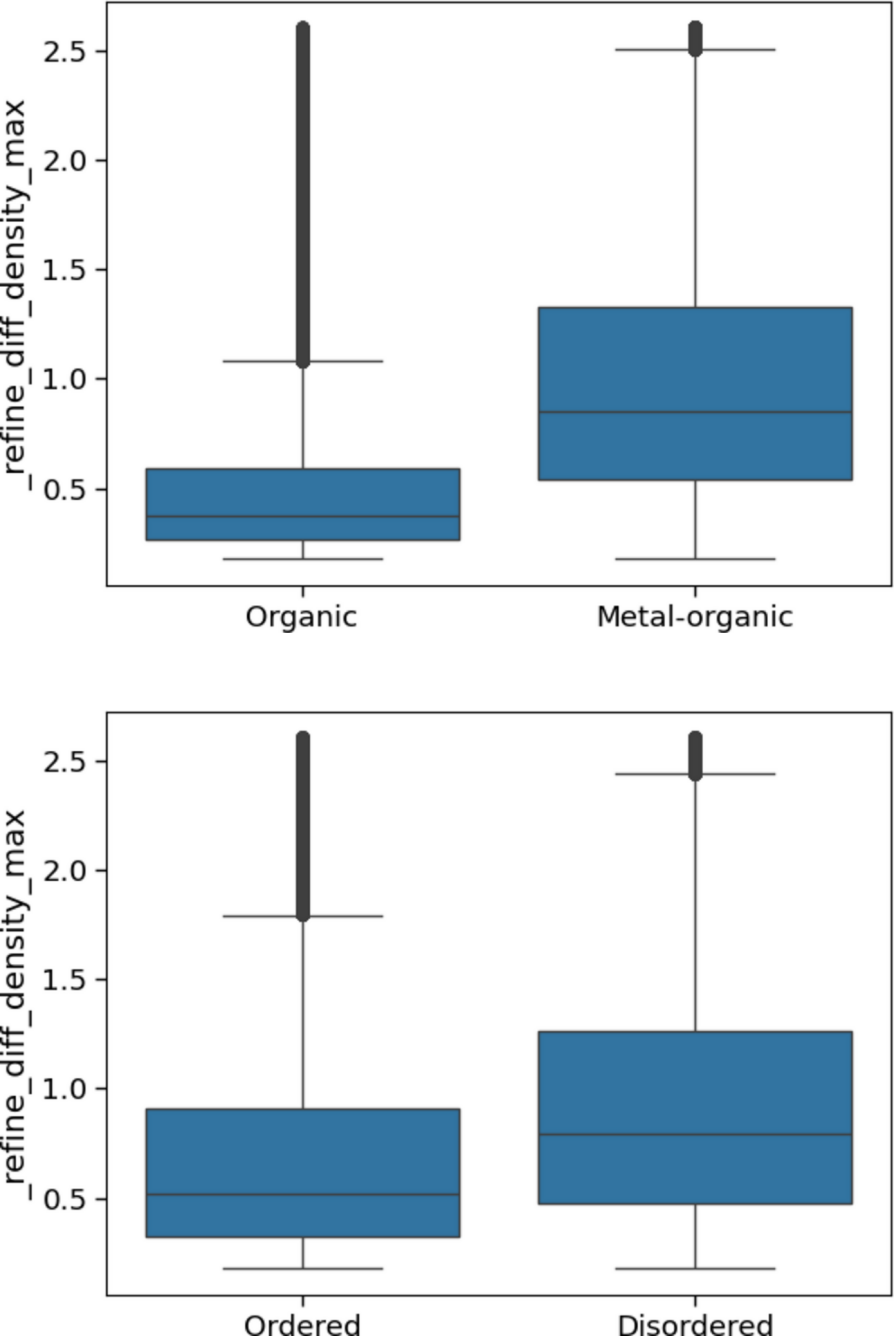
Box plots of the values of _refine_diff_density_max, comparing organic and metal–organic structures (top) and ordered and disordered structures (bottom).

Viewing the differences between different categories of structures in the CSD also shows trends that would be expected. For example, metal–organic and disordered structures have larger average values than organic and non-disordered ones (Fig. 7 ▸).

**Figure 8:**
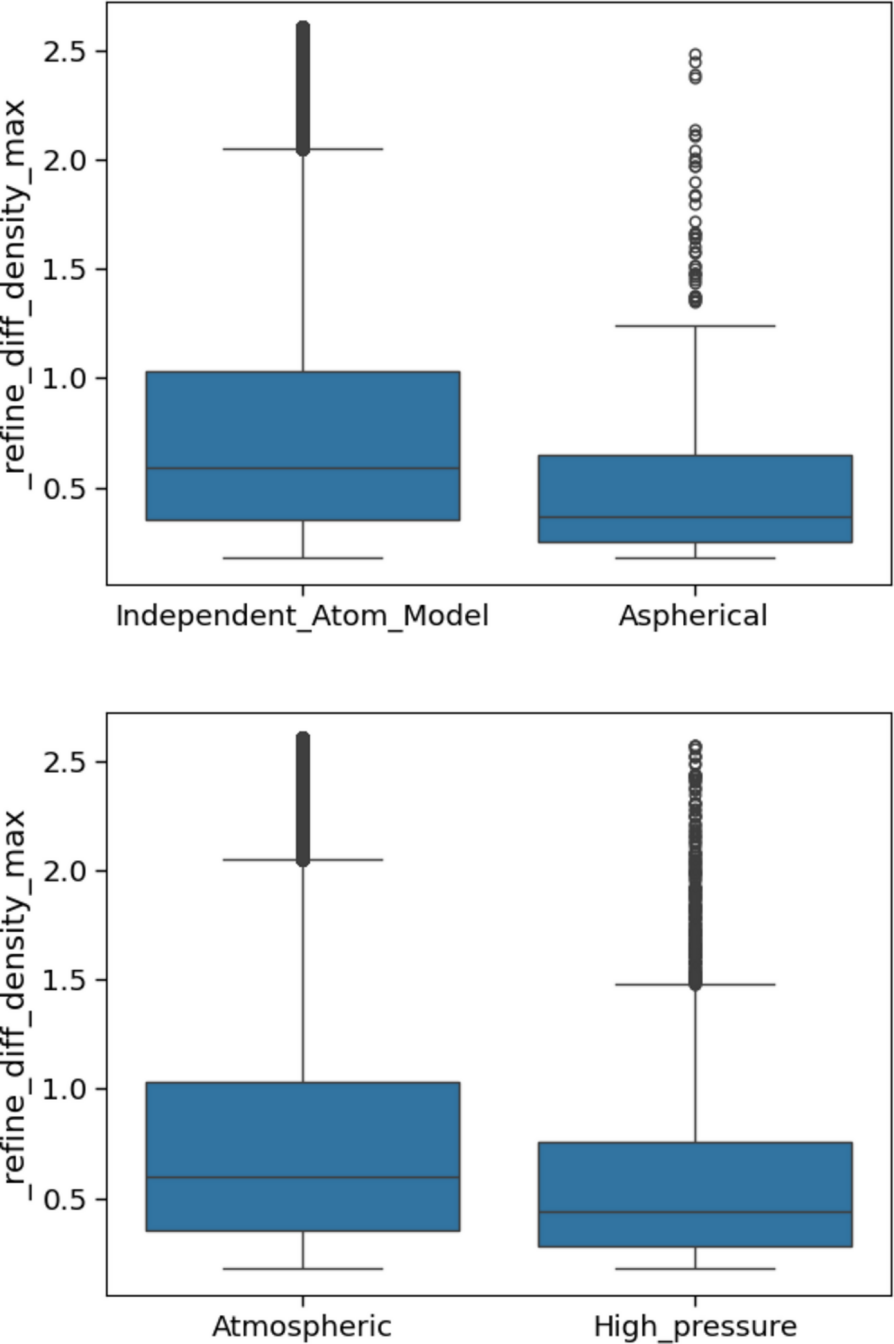
Box plots of the values of _refine_diff_density_max, comparing independent atom and aspherical atom refinement (top) and atmospheric and high pressure (bottom).

Also, as might be expected, aspherical atom refinements show smaller residual density values (Fig. 8 ▸). Slightly lower residual density values were also observed for high-pressure studies, although in these cases the datasets being compared are of very different sizes and the difference in values is not statistically significant. The motivation for carrying out these kinds of experiments is likely to be a specific investigation of some particular crystallographic features, which would further suggest that good-quality crystals and an optimal data collection routine were employed.

Before considering a more in-depth analysis of these values, the limitations of the single-point values reported for the maximum and minimum residual densities in a CIF should be noted. For example, the location of a single peak of residual density might strongly indicate an unmodelled atom, whereas if further inspection showed an area of the structure with high overall residual density, this might indicate disorder present in the structure. Likewise, the highest residual electron density might occur at or near the heaviest atoms in the refinement due to a sub-optimal absorption correction or similar systematic effects.

#### 3.2.3. Goodness of fit

The goodness of fit (GooF) is another measure of the agreement between the structural model and the measured data (Schwarzenbach *et al.*, 1989 ▸). The ideal value for the GooF is one, and the value for a structure is included in the CIF in the _refine_ls_goodness_of_fit_ref item. Across the whole CSD, both the mean and median values of goodness of fit are close to one (1.068 and 1.045, respectively), indicating a good match to the ideal value. These averages over one indicate that it is more likely for a structure to have a GooF of over one than under one. Indeed, only 15% of structures have a GooF less than one, which might arise from issues such as problems with the absorption correction when processing the data, or over-refinement, for example.

**Figure 9:**
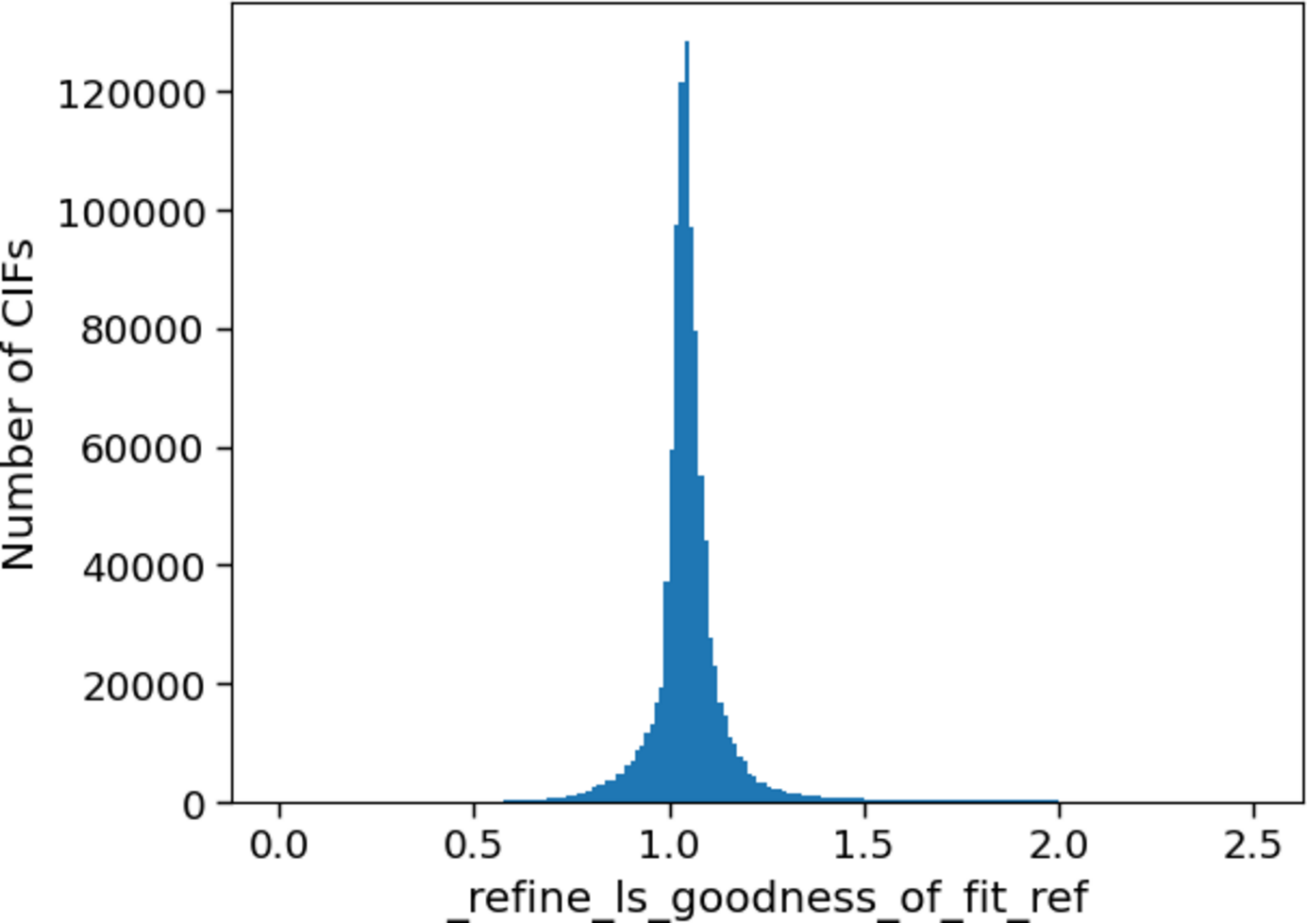
A histogram showing the values for the goodness of fit across the whole CSD.

A histogram of the spread of the data (Fig. 9 ▸) across the whole CSD shows a positively skewed distribution of values with a sharper peak than expected for a normal distribution. While there are extremes of values, 80% of the reported GooFs are between 0.969 and 1.143. The boundaries of the *checkCIF* alerts are significantly outside this range: even the level C alert, at less than 0.8 or greater than 2, covers over 96% of the CIFs analysed. This suggests that the *checkCIF* alerts for this quality metric might not provide valuable feedback to the crystallographer, and stricter limits could be selected that would be relevant for the majority of structures refined against the squared structure factors, *F*^2^. The current C alert levels of <0.8 and >2.0 might be better suited to the A or B alert boundaries (which are currently set at <0.4 or >6.0 and <0.6 or >4.0, respectively). Comparing the goodness of fits for different categories of structures showed no significant variation of values.

#### 3.2.4. Shift

An indicator of good convergence of a least-squares refinement is that the largest shift in each parameter is much smaller than its standard uncertainty (Watkin, 2008 ▸). The CIF item _refine_ls_shift/su_max is defined as ‘the largest ratio of the final least-squares parameter shift to the final standard uncertainty’ (https://www.iucr.org/__data/iucr/cifdic_html/1/cif_core.dic/Irefine_ls_shift=over=su_max.html), where the shift is the change that occurred in the values for the parameters during the last round of refinement. This value should be as small as possible. Negligible final parameter shifts should be obtained from the iterative refinement process when the calculation of new shifts from the latest parameter values is repeated (Clegg *et al.*, 2009 ▸).

**Figure 10:**
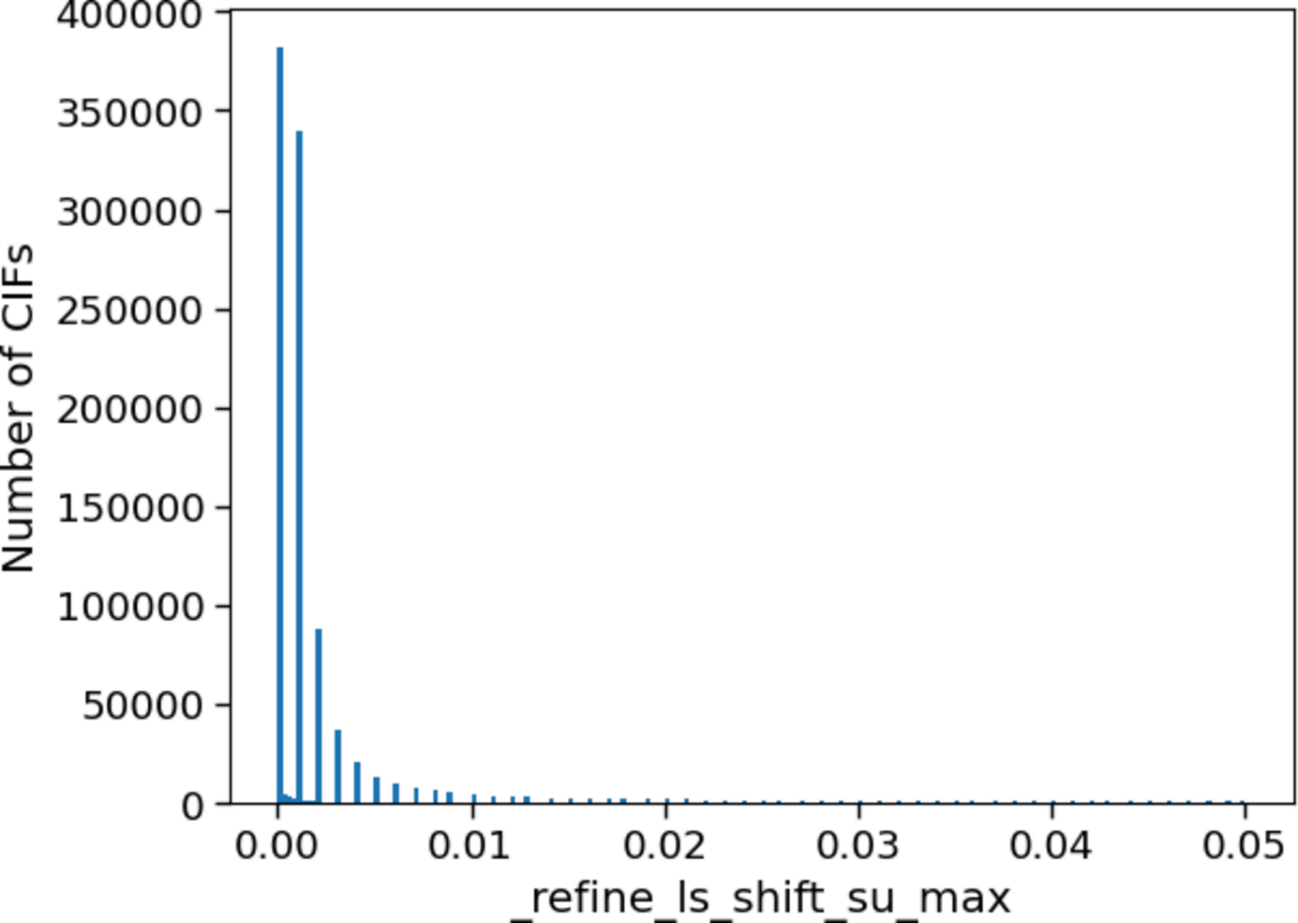
_refine_ls_shift/su_max values for crystal structures in the CSD.

A high value of _refine_ls_shift/su_max indicates that convergence has not been achieved for the refinement. This could be indicative of a poor initial model, a disordered structure or other problems that result in continual oscillation of some of the parameters (see https://journals.iucr.org/services/cif/checking/SHFSU_01.html). The parameters affected by this should be noted in the CIF item _publ_section_exptl_refinement. A further refinement cycle can usually rectify the situation, but for some structures many additional rounds of refinement might be needed. It should be noted that different refinement software packages might potentially result in different maximum shifts depending on the convergence criteria that are used. This is unlikely to have any impact on the conclusions that can be drawn from an overview of structures in the CSD. Taking the CSD as a whole, most structures have a shift close to zero (Fig. 10 ▸), with a median shift value of 0.001 and the 90th percentile for the analysed CIFs falling at 0.008. These values are below the threshold for a level C *checkCIF* alert and suggest that most structures in the CSD have converged.

Similar values are observed when the CSD is divided into different categories of structures. As can be seen from Table 3 ▸, very little difference is seen between polymeric and discrete structures, organic and metal–organic structures, ordered and disordered structures, atmospheric and high-pressure studies, and between independent atom and aspherical atom models. While the upper quartile and 90th percentile values are higher for neutron and particularly electron diffraction techniques when compared with those from X-ray diffraction, these values are still close to zero, and convergence has been achieved for the majority of structures across all categories and techniques.

#### 3.2.5. Maximum theta angle and resolution

The measure of resolution of a diffraction pattern is the smallest distance between adjacent lattice planes (*d*-spacing) that was measured by the diffractometer (Dubach & Guskov, 2020 ▸). Higher-resolution reflections relate to Miller planes with smaller *d*-spacing, therefore measuring these reflections can allow the gathering of finer detail about the distribution of electron density in the crystal structure. The maximum value of θ (or smallest distance between the measured planes) measured will depend on a number of factors, including the limitations of the equipment, the experimental conditions or the properties of the crystal.

It would be most straightforward to simply use the CIF item _diffrn_reflns_resolution_max to compare the maximum resolution of diffraction patterns. However, this value is only reported in a small fraction of CIFs in the CSD (<0.015%), so other information must be used. The resolution of the diffraction pattern can instead be calculated with the Bragg equation, using the wavelength of the radiation and the maximum θ angle for the experiment. These items are almost always included in the CIF (98% of the time for _diffrn_radiation_wavelength and 97% of the time for _diffrn_reflns_theta_max).

**Figure 11:**
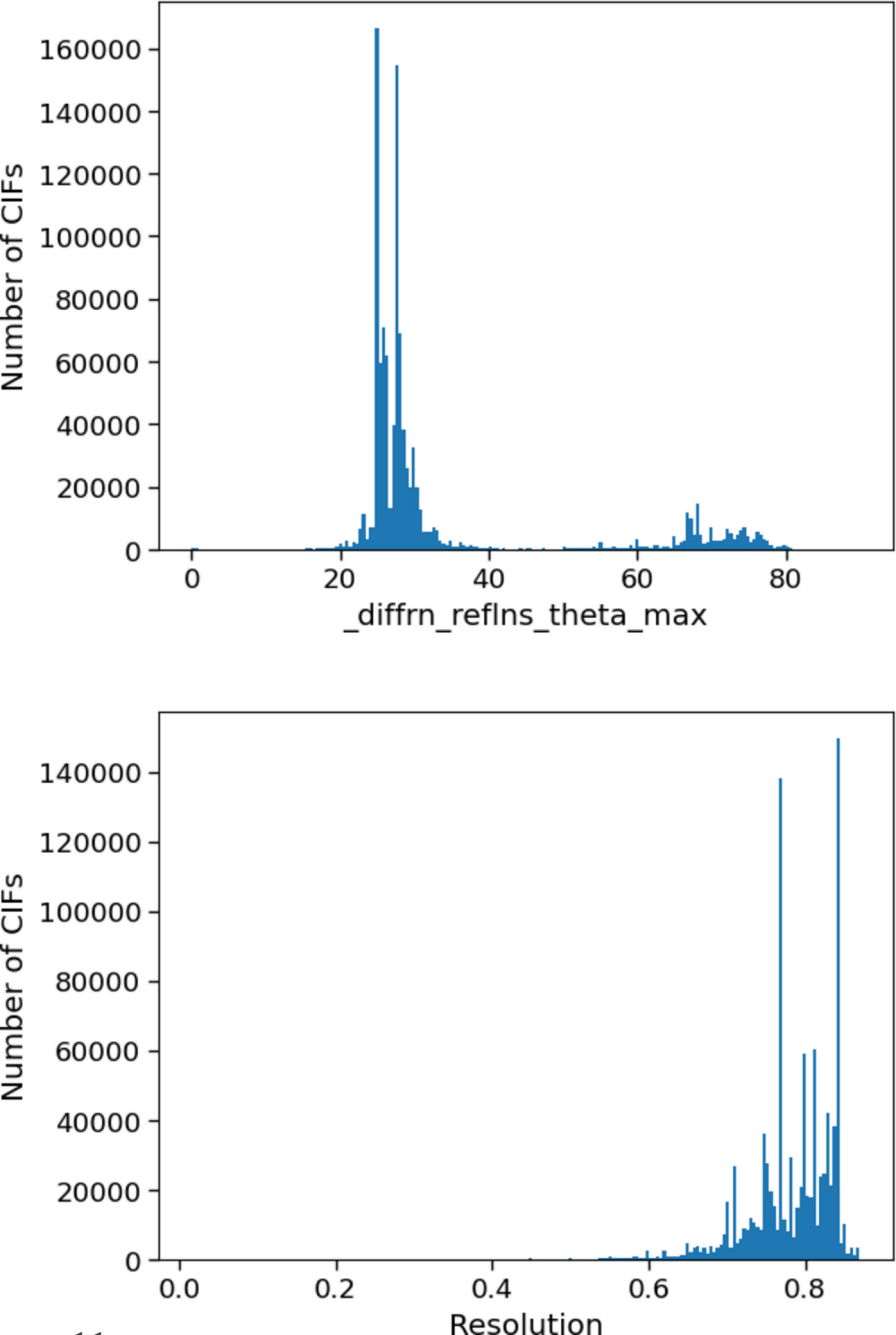
Histograms of θ_max_ values (top) and calculated resolution (Å) (bottom) for all structures in the CSD.

The item _diffrn_reflns_theta_max is the ‘maximum theta angle in degrees for the measured intensities’. In experimental terms, this is the maximum angle between the incident beam and the reflection plane in the crystal. The distribution of θ_max_ values in the CSD (Fig. 11 ▸, top) shows two separate clusters containing values around 25–35° and 65–80° (corresponding to structures measured with molybdenum and copper X-ray radiation, respectively). However, by comparison, the calculated resolution of structures in the CSD as a whole (Fig. 11 ▸, bottom) shows that the resolutions of most structures in the CSD form a single cluster at around 0.7–0.85 Å. This fits with best practice for the minimum resolution for collecting X-ray data (Spek, 2020 ▸, 2003 ▸), where *checkCIF* alerts will be raised for a resolution below 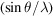 = 0.6 Å^−1^ (or greater than 0.84 Å in real space). As a number of different wavelengths are used in X-ray crystallography (Cu, Mo, Ag on a home X-ray source, tuneable for synchrotron data and very short for electron crystallography), the wavelength can make a big difference in the calculated resolution.

Across the various categories there is very little difference between the median values. Perhaps the most surprising finding is that there is no statistical difference between aspherical atom and independent atom model structures, but this could possibly be explained by large standard deviations calculated for the resolution values (Table S3) and the fact that not all aspherical atom models require high-resolution data (Sanjuan-Szklarz *et al.*, 2016 ▸).

### 3.3. Future directions

The CSD has recently passed 1.3 million individual datasets and continues to grow by over 60 000 new structures each year. Whilst this continuing growth offers the potential for exciting new insights or increased confidence in statistical analyses, it does also pose new challenges for how these crystallographic data are utilized. Developments in both crystallography and data analysis have led to the fields moving from specialist to more mainstream interest, and as such a wider cohort of users will want to assess the values associated with structure refinements. It is, therefore, perhaps incumbent upon the data providers to ensure that the user appreciates what the values mean and what they show. With an increasing reliance on artificial intelligence and machine learning to look for patterns within datasets and build predictive models, the need to effectively filter out unsuitable structures from a training set has become important – particularly because the model produced will not provide any context from the conclusions it has obtained from the training data.

In 2006 a process for determining the ‘best’ representative of each unique polymorph in the CSD was developed (van de Streek, 2006 ▸). At the time, this involved analysis of 243 355 well determined crystal structures – approximately 20% of the CSD’s current size. As the CSD continues to grow, this kind of investigation is likely to become even more relevant, as there are sufficient data for analyses to be more tailored to particular use cases. The user will want to be sure that the data being analysed are the most suitable from all the structures available, both in terms of quality and applicability.

For crystallographic databases to continue to meet the challenges and expectations of the user community, there is a potential need for additional filtering and quality-assessment tools to assist the user in selecting the most appropriate data for their requirements. The analysis performed in this article enables the user to judge an individual refinement in the context of all the similar data available to them. To achieve this on an ongoing basis, three additional developments to the CSD and the associated database search and analysis tools would be beneficial. Initially, this might require the addition of more data fields; these could be calculated ‘on the fly’ as well as new data items being extracted from the deposited CIFs, such as in this case. While some fields would be more applicable to specific areas, *e.g.* void information for frameworks, an essential criterion for adding more data fields would be helping users to find entries most applicable to their applications. Secondly, software should allow users to filter search queries based on these data fields. Since this functionality would be particularly beneficial to high-throughput or data-mining applications, this filtering could be most useful in the CSD Python API (Sykes *et al.*, 2024 ▸), as has been implemented for the examples in this work. However, as manual preparation of a dataset using tools such as *Mercury* is a common approach, GUI-based filtering options might also be appropriate in some circumstances. Finally, for individual entries, providing a high-level overview of the structural and refinement features would be a constructive approach. An excellent example of this can be found in the protein data community, with the validation report ‘slider’ graphic produced by the Protein Data Bank (Read *et al.*, 2011 ▸; Gore *et al.*, 2017 ▸). Another option would be to adopt a similar approach to that taken by the International Centre for Diffraction Data (ICDD) for powder diffraction data, where individual powder patterns are assigned ‘quality marks’. These marks, from a star to represent the highest quality pattern, to O for a poor quality pattern, give the user a way of quickly assessing whether the pattern in question is suitable for their requirements (Gates-Rector & Blanton, 2019 ▸).

The findings from investigations like those in this publication could underpin any similar venture from the CCDC, allowing a structure to be judged in a suitable context from the whole CSD. There is an important educational role too, for example in understanding that high-pressure or electron diffraction data are not ‘poor’ quality but give different values to ambient pressure X-ray diffraction data due to the experimental conditions. It would therefore be appropriate to compare certain values for high-pressure or electron diffraction data to the subset of other equivalent data, rather than to use averages taken from all structures contained within the CSD.

## 4. Conclusion

It is important to note that the values surveyed here are single-point values and might not give a full picture of the validity of any single crystal structure. *checkCIF* reports, for example, take into account many additional measures of structural validity and quality, as well as looking at how the values of quality metrics change with factors such as resolution and reflection intensity. Investigating a crystal structure in detail can involve going beyond assessing the minimum and maximum values of the electron density by looking at a difference electron density map. The article *checkCIF validation ALERTS: what they mean and how to respond* (Spek, 2020 ▸) notes, when referring to difference density maps, ‘Such a map should be close to featureless with similar positive and negative density excursions of less than ∼±0.5 e Å^−3^’. A refinement might have one single isolated disagreement between model and data leading to a high value, or many such disagreements. These two situations might lead to a similar value for _refine_diff_density_max, but the latter would suggest that the model should be treated with more caution. This further re-enforces the notion that an assessment of the quality of the data and its suitability for various use cases must involve the consideration of multiple factors.

In recent years, increased computational resources and advanced data analysis tools have led to a resurgence of interest in ‘big data’. The integrity, or veracity, of the data is an important area. It is obviously impractical to manually inspect the input for suitability of thousands or millions of fields, which increases the need for metrics for filtering and assessment. The user of such data will need to have an opinion on what is normal, and also what can be considered correct and reliable. As shown from the investigations in this article, a single ‘one size fits all’ approach is not appropriate, as different data sources and use cases lead to different acceptance criteria. For example, different techniques used for the determination of a crystal structure (‘standard’ laboratory X-ray, synchrotron X-ray, neutron or electron diffraction *etc*.) will have different ‘normal’ ranges, and this is then compounded by changes in equipment and software over time. Another important factor when considering the use of crystallographic data is interoperability, one of the FAIR principles (Wilkinson *et al.*, 2016 ▸). If a scientist is comparing protein data from the Protein Data Bank (PDB) (wwPDB consortium, 2019 ▸) to organic small-molecule data from the CSD or inorganic data from the Inorganic Crystal Structure Database (ICSD) (Zagorac *et al.*, 2019 ▸), knowing that the data are ‘equivalent’, in terms of what conclusions might be drawn from them, is important.

While the CCDC encourages the dissemination of more crystallographic data, it remains crucial that users are able to assess whether the data are ‘fit for purpose’. This reflects the diverse objectives for which crystallographic data are collected, each with different requirements for accuracy and precision. We hope that this study, along with future investigations, will support users of the CSD in making informed decisions about which data are relevant and of sufficient quality to fit their specific research needs. Fundamentally, the core questions persist: Can the data be trusted? Are they suitable for reuse? As the CSD marks its 60th anniversary, it is noteworthy that although the challenges faced by researchers have evolved, the value of comprehensive databases such as the CSD in advancing scientific inquiry remains as significant as it was in 1965.

## Supplementary Material

Supporting tables and figures. DOI: 10.1107/S2052252525007134/yc5051sup1.pdf

## Figures and Tables

**Table 1:** Data completeness, range permitted by CIF dictionary, allowed values and number of CIFs outside this range for selected items

CIF data item	Data completeness (%)	CIF dictionary permitted range	Range used in this study	Number of non-permitted values
_refine_ls_R_factor_gt	95.7	0.0 → infinity	0 < *x* < 1	279
_refine_ls_wR_factor_ref	94.4	0.0 → infinity	0 < *x* < 1	19
_refine_ls_shift/su_max	94.2	0.0 → infinity	0 < *x* < 15	1781
_refine_diff_density_max	97.3	none given	0 < *x* < 118	508
_refine_diff_density_min	97.3	none given	−118 < *x* < 0	566
_refine_ls_goodness_of_fit_ref	94.4	0.0 → infinity	0 < *x* < 20	27
_diffrn_reflns_theta_max	97.3	0.0 → 90.0	0 < *x* < 90	66

**Table 2:** Statistics for residual electron density values for the whole CSD Sample sizes are *n* = 1 056 773 and *n* = 1 056 718 for the maximum and minimum residual electron density, respectively. Absolute values for the minimum values are reported to simplify comparison of the statistics for the maximum and minimum densities.

Parameter	Variance	Standard deviation	Lower quartile	Median	Mean	Upper quartile
_refine_diff_density_max	1.116	1.056	0.336	0.596	0.904	1.109
|_refine_diff_density_min|	0.582	0.763	0.289	0.481	0.714	0.860

**Table 3:** Statistics for _refine_ls_shift/su_max values for categories of crystal structures in the CSD

Category†	Structures	Median	Upper quartile	90th percentile
X-ray diffraction	1019912	0.001	0.002	0.008
Neutron diffraction	691	0.000	0.006	0.041
Electron diffraction	390	0.001	0.037	0.184
Not disordered	702502	0.001	0.001	0.006
Disordered	318491	0.001	0.003	0.015
Non-polymeric	887949	0.001	0.002	0.009
Polymeric	133044	0.001	0.002	0.008
Organic	459591	0.000	0.001	0.006
Metal–organic	561402	0.001	0.002	0.011
Independent atom model	1019241	0.001	0.002	0.008
Aspherical atom model	1752	0.001	0.001	0.010
Atmospheric pressure	1016625	0.001	0.002	0.009
High pressure	4368	0.000	0.000	0.002

† For all categories, the minimum, 10th percentile and lower quartile are zero.

## Data Availability

Data supporting the reported results are available either within the article or in the supporting information.
