# Supplementary material for: A survey of crystallographic quality metrics from CIFs in the Cambridge Structural Database
Source: IUCrJ. 2025 Sep 22;12(Pt 6):647–57. doi: 10.1107/S2052252525007134 (PMC12573924; doi:10.1107/S2052252525007134)
Supplement: Supplementary file 1 [file m-12-00647-sup1.pdf]

IUCrJ

Volume 12 (2025)

Supporting information for article:

A survey of crystallographic quality metrics from CIFs in the Cambridge Structural Database

Clare A. Tovee, Seth B. Wiggin, Natalie T. Johnson, Philip I. Andrews and Matthew P. Lightfoot

S1. Statistical Tables

Table S1 Descriptive statistics for the seven CIF quality metrics and calculated resolution values for the entire CSD dataset examined in this study.

Field	Total	Mean	Median	StdDev	Var
_refine_ls_R_factor_gt	1039467	0.051	0.046	0.025	0.001
_refine_ls_wR_factor_ref	1025877	0.135	0.118	0.072	0.005
_refine_diff_density_max	1056773	0.904	0.595	1.056	1.116
_refine_diff_density_min	1056718	0.714	0.481	0.763	0.582
_refine_ls_goodness_of_fit_ref	1026745	1.068	1.045	0.234	0.055
_refine_ls_shift/su_max	1020993	0.042	0.001	0.437	0.191
Resolution[1]	1056173	0.794	0.799	1.278	1.633

[1] Resolution has been calculated from _diffrn_reflns_theta_max and _diffrn_radiation_wavelength CIF fields

Table S2 Descriptive statistics for the CIF field _diffrn_reflns_theta_max across all categories examined in this study.

Category	Total	Mean	Median	StdDev	Var
Electron	390	3.408	0.885	9.086	82.561
Neutron	612	56.200	59.69	19.911	396.455
X-ray	1056452	33.885	27.49	15.893	252.599
Disordered	326718	34.510	27.49	16.575	274.733
Ordered	730736	33.609	27.49	15.601	243.395
Metal-organic	580884	31.380	27.48	13.133	172.488
Organic	476570	36.943	27.52	18.294	334.685
Discrete	922119	34.338	27.5	16.283	265.120
Polymer	135335	30.817	27.21	12.711	161.566
Aspherical	1718	41.503	36.39	15.913	253.236

Independent Atom Model	1055736	33.875	27.49	15.911	253.156
Atmospheric	1053052	33.925	27.49	15.931	253.804
High pressure	4402	24.713	26.084	6.025	36.300

Table S3 Descriptive statistics for data resolution across all categories examined in this study.

Category	Total	Mean	Median	StdDev	Var
Electron	390	0.824	0.850	0.237	0.056
Neutron	419	0.646	0.673	0.220	0.048
X-ray	1055364	0.794	0.799	1.279	1.635
Disordered	326481	0.802	0.800	1.635	2.675
Ordered	729692	0.790	0.796	1.080	1.167
Metal-organic	580280	0.793	0.795	1.673	2.801
Organic	475893	0.794	0.800	0.458	0.210
Discrete	920929	0.793	0.798	1.368	1.872
Polymer	135244	0.795	0.800	0.072	0.005
Aspherical	1709	0.590	0.590	0.159	0.025
Independent Atom Model	1054464	0.794	0.799	1.279	1.636
Atmospheric	1051803	0.793	0.799	1.234	1.523
High pressure	4370	0.870	0.794	5.301	28.101

Resolution was calculated using the formula d = wavelength/2*sin(theta_max in radians). Totals differ from the _diffrn_reflns_theta_max due to availability of wavelengths from which to calculate the resolution.

Table S4 Descriptive statistics for the CIF field _refine_ls_shift/su_max across all categories examined in this study.

Category	Total	Mean	Median	StdDev	Var
Electron	380	0.150	0.0018	0.519	0.269
Neutron	691	0.103	0.0003	0.783	0.613

X-ray	1018226	0.042	0.001	0.437	0.191
Disordered	318055	0.054	0.001	0.487	0.237
Ordered	701242	0.037	0.001	0.413	0.170
Metal-organic	560522	0.052	0.001	0.490	0.240
Organic	458775	0.031	0	0.362	0.131
Discrete	886362	0.042	0.001	0.435	0.189
Polymer	132935	0.044	0.001	0.450	0.203
Aspherical	1699	0.016	0.0006	0.265	0.070
Independent Atom Model	1017598	0.042	0.001	0.437	0.191
Atmospheric	1014934	0.042	0.001	0.438	0.192
High pressure	4363	0.009	0	0.105	0.011

Table S5 Descriptive statistics for the CIF field _refine_diff_density_max across all categories examined in this study.

Category	Total	Mean	Median	StdDev	Var
Electron	382	0.353	0.2555	0.439	0.192
Neutron	674	1.311	0.9035	1.682	2.828
X-ray	1055225	0.905	0.595	1.056	1.115
Disordered	326483	1.143	0.837	1.135	1.288
Ordered	729798	0.798	0.503	1.001	1.003
Metal-organic	580296	1.248	0.919	1.227	1.505
Organic	475985	0.485	0.346	0.568	0.323
Discrete	921076	0.854	0.552	1.005	1.010
Polymer	135205	1.247	0.906	1.306	1.705
Aspherical	1664	0.430	0.25	0.599	0.359
Independent Atom Model	1054617	0.905	0.596	1.057	1.117
Atmospheric	1051929	0.906	0.597	1.058	1.119
High pressure	4352	0.549	0.34	0.637	0.406

Table S6 Descriptive statistics for the CIF field _refine_diff_density_min across all categories examined in this study.

Category	Total	Mean	Median	StdDev	Var
Electron	382	-0.336	-0.2355	0.400	0.160
Neutron	674	-1.579	-0.882	3.839	14.739
X-ray	1055106	-0.714	-0.481	0.757	0.573
Disordered	326470	-0.876	-0.634	0.835	0.698
Ordered	729692	-0.642	-0.421	0.717	0.514
Metal-organic	580261	-0.979	-0.724	0.876	0.767
Organic	475901	-0.391	-0.294	0.409	0.167
Discrete	920955	-0.671	-0.449	0.718	0.516
Polymer	135207	-1.005	-0.729	0.967	0.935
Aspherical	1664	-0.393	-0.246	0.469	0.220
Independent Atom Model	1054498	-0.715	-0.482	0.763	0.583
Atmospheric	1051810	-0.715	-0.482	0.764	0.583
High pressure	4352	-0.510	-0.3248	0.575	0.331

Table S7 Descriptive statistics for the CIF field _refine_ls_R_factor_gt across all categories examined in this study.

Category	Total	Mean	Median	StdDev	Var
Electron	392	0.194	0.178	0.074	0.005
Neutron	706	0.076	0.068	0.044	0.002
X-ray	1038369	0.051	0.046	0.025	0.001
Disordered	321376	0.059	0.053	0.028	0.001
Ordered	718091	0.048	0.044	0.023	0.001
Metal-organic	569120	0.050	0.044	0.025	0.001
Organic	470347	0.054	0.048	0.025	0.001
Discrete	905669	0.051	0.046	0.024	0.001

Polymer	133798	0.051	0.045	0.028	0.001
Aspherical	1759	0.025	0.020	0.020	0.000
Independent Atom Model	1037708	0.051	0.046	0.025	0.001
Atmospheric	1035089	0.051	0.046	0.025	0.001
High pressure	4378	0.066	0.058	0.037	0.001

Table S8 Descriptive statistics for the CIF field _refine_ls_wR_factor_ref across all categories examined in this study.

Category	Total	Mean	Median	StdDev	Var
Electron	389	0.445	0.440	0.144	0.021
Neutron	667	0.151	0.140	0.084	0.007
X-ray	1024821	0.135	0.118	0.072	0.005
Disordered	319176	0.159	0.140	0.082	0.007
Ordered	706701	0.125	0.111	0.065	0.004
Metal-organic	562744	0.129	0.111	0.072	0.005
Organic	463133	0.143	0.126	0.072	0.005
Discrete	892762	0.135	0.119	0.071	0.005
Polymer	133115	0.136	0.115	0.079	0.006
Aspherical	1550	0.051	0.038	0.054	0.003
Independent Atom Model	1024327	0.135	0.119	0.072	0.005
Atmospheric	1021536	0.135	0.118	0.072	0.005
High pressure	4341	0.170	0.142	0.106	0.011

Table S9 Descriptive statistics for the CIF field _refine_ls_goodness_of_fit_ref across all categories examined in this study.

Category	Total	Mean	Median	StdDev	Var
Electron	389	1.774	1.466	1.040	1.082
Neutron	674	1.474	1.146	1.042	1.086

X-ray	1025682	1.067	1.045	0.231	0.053
Disordered	319183	1.072	1.046	0.210	0.044
Ordered	707562	1.066	1.045	0.244	0.060
Metal-organic	563175	1.065	1.045	0.205	0.042
Organic	463570	1.071	1.045	0.265	0.070
Discrete	893631	1.067	1.044	0.238	0.057
Polymer	133114	1.074	1.052	0.206	0.043
Aspherical	1691	1.432	1.1579	1.033	1.067
Independent Atom Model	1025054	1.067	1.045	0.230	0.053
Atmospheric	1022402	1.067	1.045	0.234	0.055
High pressure	4343	1.133	1.089	0.252	0.063

S2. Data completeness

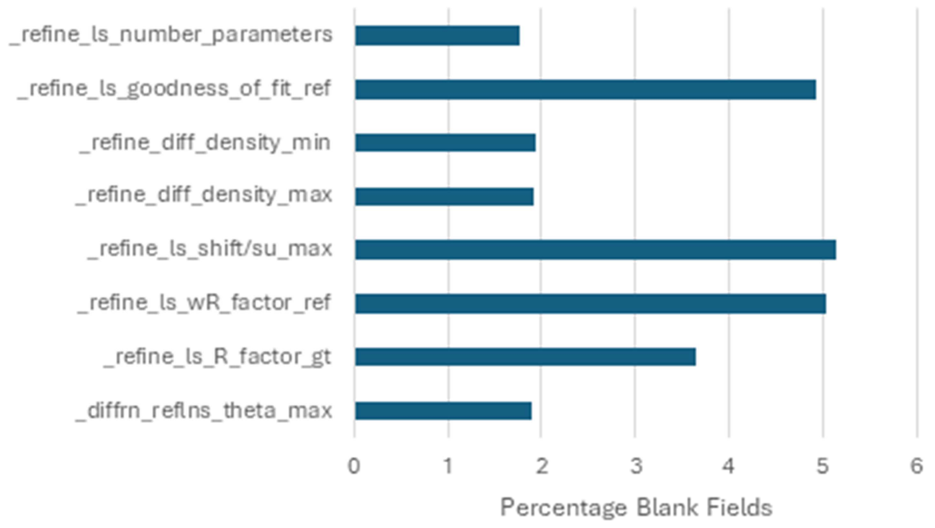

Figure S1 Bar chart of percentage of CIFs missing the field (excluding constraints)

S3. Constraints, Restraints and Parameters

Looking at the refinement statistics for the number of parameters, restraints and constraints used also shows interesting differences. As would be expected, disordered structures involve more parameters and restraints on the refinement, however there is also a significant reduction in the average number of constraints applied. This perhaps shows how crystallographers tackle challenges with disorder, by first trying to apply restraints, and then resorting to more complex approaches when required.

Table S10 Descriptive statistics for the CIF field _refine_ls_number_restraints across all categories examined in this study.

Category	Total	Mean	Median	StdDev	Var
Electron	390	303.0	68	1302.2	1695864.3
Neutron	726	104.8	0	615.8	379196.1
X-ray	1035125	78.0	0	627.5	393734.9
Disordered	322635	213.6	12	1064.1	1132399.4
Ordered	713606	16.9	0	220.1	48437
Metal-organic	569945	105.1	0	751.7	565101.6
Organic	466296	45.2	0	428.3	183412.2
Discrete	902082	79.6	0	656.9	431569.1
Polymer	134159	68.0	0	378.3	143079.5
Aspherical	1813	13.0	0	84.6	7156.9
Independent Atom Model	1034428	78.2	0	628.4	394905.3
Atmospheric	1031871	78.1	0	629.1	395773
High pressure	4370	85.7	21	175.7	30864.5

Data from 88 CIFs which appeared to have an erroneously high number of restraints (>50,000) were excluded as outliers

Table S11 Descriptive statistics for the CIF field _refine_ls_number_constraints across all categories examined in this study.

Category	Total	Mean	Median	StdDev	Var
Electron	70	47.943	5	72.484	5253.997
Neutron	89	2.101	0	9.401	88.383
X-ray	25235	21.611	0	74.465	5544.982
Disordered	5697	32.210	0	104.536	10927.819
Ordered	19697	18.551	0	62.648	3924.737
Metal-organic	12897	22.419	0	65.996	4355.472
Organic	12497	20.786	0	82.081	6737.315
Discrete	23017	21.520	0	75.746	5737.437
Polymer	2377	22.542	0	59.180	3502.247

Aspherical	1061	13.422	0	45.303	2052.385
Independent Atom Model	24333	21.973	0	75.345	5676.808
Atmospheric	25283	21.660	0	74.506	5551.193
High pressure	111	11.559	11	14.580	212.589

Table S12 Descriptive statistics for the CIF field _refine_ls_number_parameters across all categories examined in this study.

**Category**	**Total**	**Mean**	**Median**	**StdDev**	**Var**
Electron	392	262.189	172.5	361.948	131006.214
Neutron	771	379.355	244	418.820	175409.923
X-ray	1057569	412.318	316	362.136	131142.731
Disordered	327005	581.142	454	508.116	258182.373
Ordered	731727	336.757	277	236.606	55982.567
Metal-organic	581617	470.092	372	399.772	159817.851
Organic	477115	341.714	264	295.338	87224.729
Discrete	923315	422.125	322	369.238	136336.917
Polymer	135417	344.828	271	301.396	90839.516
Aspherical	1839	320.631	216	308.790	95351.321
Independent Atom Model	1056893	412.398	316	362.259	131231.768
Atmospheric	1054356	413.432	316	362.372	131313.532
High pressure	4376	124.615	87	130.059	16915.301

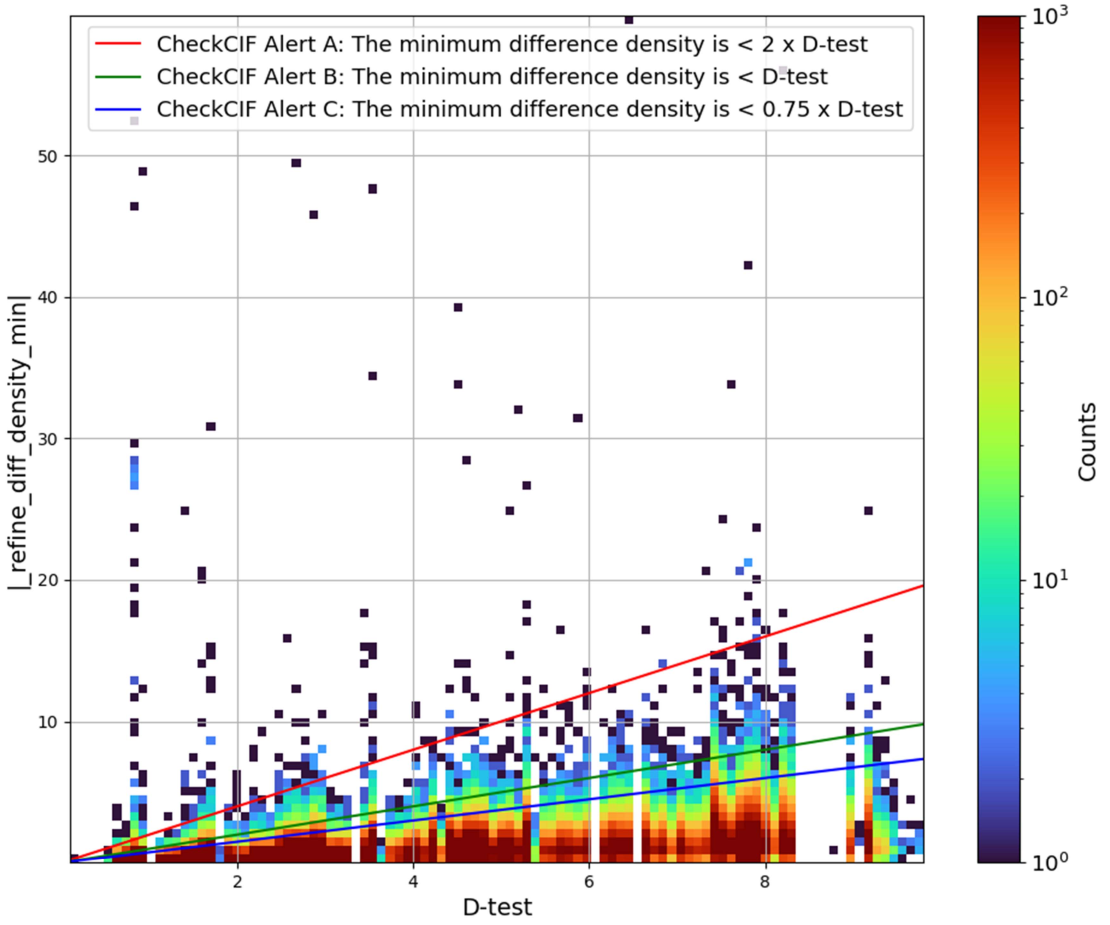

Figure S2 Scatter plot of the checkCIF D-test value against |_refine_diff_density_min| with a logscale for the colour indicating the number of structures within each binned gridpoint.
